# HAART, DOTS and renal disease of patients co-infected with HIV/AIDS and TB in the South West Region of Cameroon

**DOI:** 10.1186/s12889-015-2331-z

**Published:** 2015-10-09

**Authors:** Dickson Shey Nsagha, Benjamin Thumamo Pokam, Jules Clement Nguedia Assob, Anna Longdoh Njunda, Odette Dzemo Kibu, Elvis Asangbeng Tanue, Charlotte Wenze Ayima, Patrick Elroy Weledji

**Affiliations:** Department of Public Health and Hygiene, Faculty of Health Sciences, University of Buea, P.O Box 12, Buea, Cameroon; Department of Medical Laboratory Sciences, Faculty of Health Sciences, University of Buea, P.O Box 12, Buea, Cameroon; Department of Surgery, Obstetrics and Gynaecology, Faculty of Health Sciences, University of Buea, P.O Box 12, Buea, Cameroon

**Keywords:** HIV/AIDS, TB, HAART, DOTS, Renal, Cameroon

## Abstract

**Background:**

Tuberculosis is the commonest infection among HIV/AIDS patients. This co-infection constitutes a major death threat in the world. There is paucity of data about renal disease amongst patients on HAART and DOTS therapy in Cameroon.

**Methods:**

This was a hospital-based cross-sectional study in the Buea, Limbe and Kumba government Hospitals. Spectrophotometric method was used for the quantitative determination of serum creatinine, urea, albumin and total protein levels. Glomerular filtration rate was estimated using the MDRD method. The student’s *t* test, ANOVA and logistic regression were used to analyse the data.

**Results:**

Out of 200 participants, 101 (50.5 %) were males. The ages ranged from 21 to 65 years with a mean age of 38.04 ± 10.52 years. Compared to adults on DOTS alone, adults on HAART alone had a significantly higher prevalence of eGFR <60 ml/min/1.73 m^2^ (10/70 (14.3 %) vs. 1/70 (1.4 %), OR = 11.5 [1.4–92.5], *p* = 0.02) while more participants on HAART/DOTS had significantly higher serum creatinine (18/60 (30 %) vs 10/70 (14.3) OR = 2.57 [1.08–6.12], *p* = 0.033). Though participants on HAART/DOTS combined therapy had low eGFR, the association was not statistically significant (OR = 6.27, 95 % CI;0.71–55.27, *p* = 0.098). Participants on the Zidovudine, Lamivudine, Nevirapine regimen showed a statistically significant difference in the mean serum creatinine and albumin levels between the HAART/DOTS combined therapy and HAART group (*p* = 0.0219 and 0.0001 respectively).

**Conclusion:**

Compared to adults on DOTS, adults on HAART were more likely to have renal dysfunction (eGFR <60 ml/min per 1.73 m^2^). Adult on a combination of HAART and DOTS had a similar prevalence of renal dysfunction as those on HAART alone. This study showed that the use of the HAART regimen (Tenofovir, Lamivudine and Efavirenz combination) among the HAART treated adults was nephrotoxic. However, other combined HAART and DOTS regimens had no nephrotoxic effect. Abnormal kidney function can be associated with HAART use.

## Background

Worldwide, tuberculosis (TB) is the commonest opportunistic infection affecting 40 % of people living with HIV/AIDS (PLWHA) and it remains the commonest cause of death in patients with AIDS. Highly Active Antiretroviral Therapy (HAART) is a combination of antiretroviral drugs for the management of HIV/AIDS [1]. Directly Observed Therapy, Short-course (DOTS), is the recommended therapy for TB treatment by the World Health Organization (WHO). This is a combination of isoniazid, rifampin, pyrazinamide and ethambutol for 8 weeks, followed by isoniazid and rifampin for a further 4–7 months (standard therapy) [2].

The association between HIV and renal disease was reported in 1984 by investigators in New York and Miami, on a series of HIV-1 seropositive patients who developed a renal syndrome characterized by progressive renal failure and proteinuria [3]. HIV-1 infects renal cells and the kidney may be an important long term reservoir for the virus. Problems with kidney function in HIV infected people may be due to medications or the HIV itself. These can range from minor changes in fluid and electrolyte balance to end-stage renal disease requiring dialysis [4]. Patients on anti-tuberculosis treatment may develop acute kidney injury (AKI), but little is known about the renal outcome and prognostic factors. A study by Chai-Hao and colleagues [5] showed that 99 out of 1394 (7.1 %) patients on anti-TB treatment had AKI. Sixty (4.3 %) developed AKI within two months of anti-TB treatment, including 11 (0.7 %) with a prior history of rifampin exposure. Thirty (2.2 %) had co-morbid chronic kidney disease or end-stage renal disease.

Worldwide, patients with TB and HIV co-infection are estimated to be over one million [6]. The burden of this co-infection is particularly high in sub-Saharan Africa [6]. In Cameroon the prevalence of TB among HIV-infected adults was 38 % in 2013 [7]. Tuberculosis and HIV/AIDS have been closely linked since the emergence of AIDS [8].

Treatment of patients co-infected with HIV and tuberculosis increase the rate of mortality due to drug interaction, overlapping toxicity and side effects [9, 10]. Assessing the effect of this combined treatment on kidney function is not routinely done in hospitals in Cameroon. Hence, patients receiving this combined treatment may slowly develop kidney failure if they are not properly managed and monitored for any nephrotoxic effect of the combined therapy. These patients may end up having kidney failure or worse still lose their kidney in the long run. A kidney transplant will not be an easy option since it is very expensive and it is not done in Cameroon. Patients may take the option of hemodialysis which is also time consuming and expensive. Kidney function seems to be altered in patients receiving HIV and TB treatment. The study therefore, investigated the association between HAART, DOTS and kidney function of these patients in order to determine any nephrotoxic effects associated with such management.

## Methods

### Study design and setting

This was a hospital-based cross-sectional study carried out between April and July 2014 at the Buea, Limbe and Kumba government Hospitals. These are state-owned hospitals in the South-Western Region of Cameroon. These hospitals accommodate patients from other areas of the South-Western Region, providing health services to more than 40,000 inhabitants from this part of the country. A time limited sampling technique was used, where patients were consecutively recruited throughout the study period.

### Participants

Participants involved patients who visited the HIV/AIDS and TB treatment centers of the Buea, Limbe and Kumba government Hospitals. Concerned participants were those on HAART, DOTS or on the combined therapy (HAART/DOTS). Two hundred participants were enrolled into the study and they all satisfied the inclusion criteria.

### Inclusion and exclusion criteria

Participants on HAART, DOTS and combined HAART and DOTS were enrolled into the study**.** Adults who were 21 years of age and above were included. This is because according to the Cameroon code for the classification of adults, adulthood begins at the age of 21 years. Also, studies have shown HIV and TB co-infection to be the commonest cause of death among adults in many countries [11]. However, we excluded participants with known renal disease or pregnancy.

### Sample size determination

The prevalence of kidney disease was estimated to be 10 % using the formula for sample size calculation [12].

$n=\frac{(z)^2p\left(1-p\right)}{e^2}$ Where *n* = sample size, Z = constant = 1.96, *p* = Estimated prevalence = 10 %, e = precision of the event of interest = 0.05 $n=\frac{(1.96)^2(0.1)(0.9)}{0.05^2}$ = 138 participants.

### Data collection

A structured questionnaire was administered to each participant to obtain socio-demographic characteristics and medical history. Hospital records of the participants were also reviewed to obtain information about the type of drug regimen and duration of treatment.

### Laboratory procedures

Blood samples were collected from each participant through venipuncture using a vacutainer needle. About 5 ml of venous blood was collected in plain tubes. The blood samples were allowed to clot at room temperature before centrifuged at 3000 rpm for 5 min to obtain the serum. These sera were stored at a temperature of -20 °C and the measurements of serum creatinine, urea, albumin and total protein were analysed later in batches using a MINDRAY spectrophotometer. This is a BA-88A semi-auto chemistry analyzer with a touch-screen and pop-up keypad. This analyzer supports bi-chromatic tests for end point, fixed-time and kinetics methods.

For serum creatinine, the Jaffe kinetic method was used [13] while urea was analysed using the kinetic method [14]. The colorimetric method was used for the quantitative determination of serum albumin [15] and serum total protein [16]. The INMESCO kit was used for serum creatinine, urea, albumin and total protein. The estimated glomerular filtration rate (eGFR) was calculated using the Modification of Diet in Renal Disease (MDRD) equation considering the ethnic factor for the black race [17].

The study classified kidney disease based on the Kidney Disease Improving Global Outcomes (KDIGO) guidelines [18]. Decreased Renal function was defined as eGFR <60 ml/min per 1.73 m^2^ and no known kidney disease when eGFR ≥60 ml/min per 1.73 m^2^ and a stable serum creatinine. Reference ranges for renal function tests were set as follows; serum creatinine: 0.6–1.1 mg/dl, serum urea: 10–50 mg/dl, serum albumin: 35–52 g/l, serum total protein: 66–86 g/l. The participants on DOTS were set as the reference group. In the logistic regression analysis for factors associated with abnormal laboratory parameters, a serum creatinine of >1.1 mg/dl, eGFR of <60 ml/min per 1.73 m^2^, serum albumin of >52 g/l and serum total protein of > 86 g/l were considered abnormal.

### Data management and analysis

Data obtained from each participant was entered into a log book and this was checked by the lead author. The data was de-identified prior to analysis and later keyed into a computer using Microsoft excel, 2010 (Microsoft Corporation Inc. USA) and verified for the possibility of entering errors. Statistical analysis was conducted using STATA version 11. Student’s t-test was used to compare the differences between group means. Logistic regression analysis was used to determine the association between the treatment groups and kidney function tests. ANOVA was used to determine the difference in proportion amongst the three treatment groups. Statistical significance was set at *p* < 0.05 at 95 % confidence interval [12].

### Ethical approval

This study was approved by the Faculty of Health Sciences Institutional Review Board (FHSIRB) of the University of Buea, Cameroon (reference number: 2014-02-0199). A signed written consent form was obtained from each participant before enrollment into the study.

## Results

A total of 216 patients were contacted but only 200 were consecutively recruited during the study period. These 16 were excluded because they provided incomplete information with regard to medical history and demographic characteristics such as age. Baseline characteristic of the study population is described in Table 1. Out of two hundred adults enrolled into the study, 87 (43.5 %) were from the Buea Regional Hospital, 62 (31.0 %) from the Limbe Regional Hospital and 51 (25.5 %) from the Kumba District Hospital. Out of the 200 participants, 119 (59.5 %) were from the urban areas while 81 (40.5 %**)** were from the rural areas. Overall, there were 101 (50.5 %) male participants and 99 (49.5 %) females. The ages ranged from 21–65 years with the mean age being 38.04 (±10.52) years. The 200 participants were divided into three treatment groups. Sixty (30 %) participants were on HAART and DOTS combined treatment, 70 (35 %) were on HAART and 70 (35 %) were on DOTS.

**Table 1:** Baseline characteristics of the study population

		Frequency no (%)
Study Area	Buea	87 (43.5 %)
	Limbe	62 (31.0)
	Kumba	51 (25.5)
	Total	200 (100)
Gender	Male	101 (50.5)
	Female	99 (49.5)
	Total	200 (100)
Age group (years) mean = 38.04 ± 10.52 years		
	21–30	55 (27.5)
	31–40	76 (38.0)
	41–50	43 (21.5)
	≥51	26 (13.0)
	Total	200 (100)
Education level	No formal education	12 (6.0)
	Primary	87 (43.5)
	Secondary	74 (37.0)
	Tertiary	27 (13.0)
	Total	200 (100)
eGFR (ml/min/1.73 m^2^)	<60	16 (8.0)
	≥60	184 (92.0)
	Total	200 (100)
Treatment groups	HAART	70 (35.0)
	DOTS	70 (35.0)
	HAART/ DOTS	60 (30.0)
	Total	200 (100)
Type of HAART	AZT/3TC/NVP	46 (65.7)
1 HAART group (*n* = 70)	AZT/3TC/EVP	3 (4.3)
	TNV/3TC/NVP	21 (30.0)
2 HAART/DOTS group (*n* = 60)	AZT/3TC/NVP	34 (56.7)
	AZT/3TC/EVP	3 (5.0)
	TNV/3TC/NVP	23 (38.3)
	Total	130 (65)
Type of DOTS		
1 DOTS group (*n* = 70)		
	RIF/INH/ETH/PZA	38 (54.3)
	RIF/INH	32 (45.7)
2 HAART/DOTS group (*n* = 60)	RIF/INH/ETH/PZA	25 (41.7)
	RIF/INH	35 (58.3)
	Total	130 (65)

Key: *AZT/3TC/NVP* Zidovudine, Lamivudine, Nevirapine, *AZT/3TC/EFV* Zidovudine, Lamivudine, Efavirenz, *TDF/3TC/EFV* Tenofovir, Lamivudine, Efavirenz, *HAART* Highly Active Antiretroviral Therapy, *DOTS* Direct Observed Therapy, Short-course, *RIF/INH/ETH/PZA* rifamycin, isoniazid, ethambutol, pyrazinamide, *RIF/INH* rifamycin, isoniazid

Out of 130 participants on HAART and HAART/DOTS, a total of 80 (61.54 %) were of the combination of Zidovudine, Lamivudine, and Nevirapine; 6 (4.62 %) on Zidovudine, Lamivudine, and Efavirenz while 44 (33.85 %) on Tenofovir, Lamivudine, and Efavirenz. Regarding the 130 participants on DOTS and HAART/DOTS, a total of 63 (48.5 %) were on the drug regimen rifamycin, isoniazid, ethambutol, pyrazinamide (intensive phase) and 67 (51.5 %) were on rifamycin, isoniazid (continuation phase).

Table 2 shows the prevalence of decreased renal disease in the three treatment groups. Participants on HAART only, had the highest prevalence 10 (14.3 %) of decreased renal function, followed by the HAART/DOTS combined group 5 (8.3 %) and the DOTS group had the lowest 1 (1.4 %). However, the overall prevalence in the three treatment groups was 16 (8.0 %). Participants on HAART treatment for more than 2 years had a lower prevalence of decreased renal function 4/33 (12 %) compared to those on HAART treatment for ≤ 2 years 6/37 (16 %) (*p* = 0.6260).

**Table 2:** Prevalence of decreased renal function by the KDIGO definition

Parameter	HAART group *n* (%)	DOTS group *n* (%)	HAART/DOTS group *n* (%)	Total *n*(%)	*p* = value
eGFR					
<60 ml/min/1.73 m^2^	10 (14.3)	1 (1.4)	5 (8.3)	16 (8.0)	0.02
≥60 ml/min/1.73 m^2^	60 (85.7)	69 (98.6)	55 (91.7)	184 (92.0)	

Key: *eGFR* estimated Glomerular Filtration Rate, *DOTS* Direct Observed Therapy Short-course, *HAART* Highly Active Antiretroviral Therapy

Logistic regression showed that significantly more participants on HAART had low eGFR than those on DOTS alone (*p* = 0.022). Also, significantly more participants on HAART/DOTS recorded high serum creatinine than those on DOTS, with a 95 % CI odds ratio of 2.57 (1.08–6.12) (*p* = 0.033). Serum albumin of 53 g/l and higher was significantly associated with HAART treatment (OR = 11.77, 95 % CI: 2.6–53.0, *p* = 0.001). Being on HAART/DOTS therapy was significantly associated with high levels of serum total protein (*p* = 0.023), with a 95 % CI odds ratio of 2.41 (1.13–5.14) (Table 3).

**Table 3:** Logistic regression of serum creatinine, eGFR, albumin, and total protein

Parameter	Number examined	No (%)	COR	95 % CI	*p*- value
Creatinine					
DOTS only*	70	10(14.3 %)	1	-	-
HAART only	70	17(24.3 %)	1.92	0.81–4.56	0.138
HAART/DOTS	60	18(30.0 %)	2.57	1.08–6.12	0.033
eGFR					
DOTS only*	70	1(1.4)	1	-	-
HAART only	70	10(14.3)	11.50	1.43–92.47	0.022
HAART/DOTS	60	5(8.3)	6.27	0.71–55.27	0.098
Albumin					
DOTS only*	70	2(2.9)	1	-	-
HAART only	70	18(25.7)	11.77	2.6–53.0	0.001
HAAT/DOTS	60	1(1.7)	0.58	0.05–6.52	0.656
Protein					
DOTS only*	70	16(22.9)	1	-	-
HAART only	70	25(35.7)	1.88	0.89–3.94	0.097
HAART/DOTS	60	25(41.7)	2.41	1.13–5.14	0.023

Key**:** *Reference group, *COR* Crude Odd Ratio, *eGFR* estimated Glomerular Filtration Rate, *DOTS* Direct Observed Therapy, Short-course, *HAART* Highly Active Antiretroviral Therapy. Logistic regression

Table 4 shows the bi-variable analysis used to estimates the impact of HIV, HAART and DOTS status to renal function parameters. Participants on HAART had a significantly higher risk of low eGFR than those on DOTS alone even after adjusting for the differences in HAART, HAART/DOTS therapy and age (aOR = 3.5 [1.1-11.3]). Further adjustment for gender and BMI did not change these estimates. Also, more participants had significantly higher risk of high serum creatinine and high serum total protein even after adjusting the differences in HAART, HAART/DOTS, age, BMI and hypertension (aOR = 11.9 [3.32–42.54] and OR = 6.0 [1.77–19.98] respectively).

**Table 4:** Association of HAART and other variables with renal function paramaters

	eGFR	*P*-value	Serum creatinine	*P*-value	Serum albumin	*P*-value	Serum total protein	*P*-value
HAART	6.9 (0.79–59.5)	0.080	2.5 (0.94–6.43)	0.064	12.8 (2.74–59.57)	0.001	2.0 (0.92–4.37)	0.079
Adjusted for HAART + HAART/DOTS	4.6 (0.49–42.2)	0.181	2.6 (0.97–6.72)	0.058	0.58 (0.5-6–71)	0.664	2.1 (0.97–4.71)	0.060
Adjusted for HAART + HAART/DOTS + Age (>40 years)	3.5 (1.1–11.3)	0.036	1.1 (0.52–2.40)	0.775	0.36 (0.11–1.24)	0.107	1.7 (0.88–3.22)	0.113
Adjusted for HAART + HAART/DOTS + Age (>40 years) + Gender (female)	6.0 (1.41–25.61)	0.015	0.7 (0.35–1.63)	0.488	0.95 (0.34–2.67)	0.931	1.4 (0.75–2.74)	0.270
Adjusted for HAART + HAART/DOTS + Age (>40 years) + Gender (female) + BMI (≥25 kg/m^2^)	4.8 (1.48–15.35)	0.009	2.2 (1.0–4.85)	0.050	1.01 (0.31–3.27)	0.982	0.7 (0.35–1.59)	0.449
Adjusted for HAART + HAART/DOTS + Age (>40 years) + Gender (female) + BMI (≥25 kg/m^2^) + Hypertension	3.9 (0.56–26.63)	0.170	11.9 (3.32–42.54)	<0.001	1.42 (0.14–14.53)	0.770	6.0 (1.77–19.98)	0.004

Key: *BMI* body mass index, *DOTS* Direct Observed Therapy, Short-course, *HAART* Highly Active Antiretroviral Therapy, *Reference group* participants on DOTS

The mean eGFR was significantly higher in patients on HAART than those on HAART/DOTS with respect to Zidovudine, Lamivudine, Nevirapine combination [126.0 (±83.44) and 95.38 (±26.51), *p* = 0.039]. Also, the mean serum albumin was significantly higher in patients on HAART alone than those on HAART/DOTS with respect to Zidovudine, Lamivudine, Nevirapine combination [49.26 (±5.76) and 38.07 (±8.95), *p* = 0.001]. No significant difference was found in mean serum creatinine between patients on HAART and those on HAART/DOTS with respect to Tenofovir, Lamivudine, Efavirenz combination. However, patients on HAART had abnormal higher value of mean serum creatinine compared to those on HAART/DOTS [2.06 (±4.16) and 0.97 (±0.28), *p* = 0.217].

No significant difference was found in mean serum creatinine between patients on DOTS and those on HAART/DOTS at the intensive phase of TB treatment [0.97 (±0.24) and 0.95 (±0.22), *p* = 0.752]. Also, no significant difference was observed in mean serum creatinine between patients on DOTS alone and those on HAART/DOTS at the continuation phase of TB treatment [1.04 (±0.17) and 1.09 (±0.33), *p* = 0.443]. The mean serum total protein was significantly higher in patients on HAART/DOTS than those on DOTS at the continuation phase of TB treatment [86.84 (±15.66) and 78.84 (±8.65) *p* = 0.016].

## Discussion

Tuberculosis is the commonest associated opportunistic infection among HIV infected individuals. This increases the mortality rate in PLWHA. However, HAART and DOTS are currently being used to fight these infections [19].

In our study, HIV-infected adults being treated with HAART alone, were about 12 times more likely to develop low levels of eGFR compared to HIV negative TB-infected adults on DOTS therapy. Our findings are similar to the study carried out by Owiredu and colleagues [20]. They proved that 11 % of the participants on HAART had low eGFR values. However, Msango and colleagues [21] found a higher prevalence of renal dysfunction among HIV patients starting antiretroviral therapy in Tanzania. Gallant and colleagues [22] demonstrated that participants on HAART had a significant decline in eGFR especially those on tenofovir and protease inhibitors. This finding from our study might be due to the nephrotoxic effect of HAART and the duration of HAART use. Serum creatinine was also statistically significantly higher in the HAART group compared to the DOTS group. Mainasara and colleagues [23] showed that, the level of serum creatinine was significantly high among participants on HAART than those not on HAART. The result of this study was contrary to the findings of Kamga and colleagues [24] who reported that the mean serum creatinine was significantly higher in the antiretroviral treatment (ART) naïve group when compared to those who were already on ART.

Critically studying the different HAART regimens, the mean serum creatinine and eGFR of Zidovudine, Lamivudine, Nevirapine combination were within the normal range with a statistical significant difference between the HAART group and combined HAART/DOTS group. Our findings were contrary to a study carried out by Lidia and colleagues who observed a variety of HAART-related renal side effects associated differently to ARV classes or single drugs [25]. Another study showed that, a more serious but less common side effect of Zidovudine, Lamivudine, Nevirapine is the damage to the liver, severe rash and a condition called lactic acidosis, which may cause pancreatitis, or renal failure. However, HAART interacts with rifampicin [26]. Rifampicin (member of rifamycin class), a key component of short-course chemotherapy for TB, is a potent inducer of the cytochrome P450 enzyme system that metabolizes several drugs including non-nucleoside reverse transcriptase inhibitor (NNRTI) antiretroviral agents [27]. Cytochrome p450 increases the metabolism of NNRTI and their metabolites are excreted in urine. Thus, due to drug- drug interaction between DOTS and HAART, the therapeutic levels of these NNRTIs are greatly reduced in plasma.

For the participants on HAART (Tenofovir, Lamivudine, Efavirenz), the mean serum creatinine was higher above normal compared to the HAART/DOTS participants. This is line with a study that have shown that, among nucleoside reverse transcriptase inhibitors, Tenofovir (TDF) is associated with renal adverse effects [25]. TDF toxicity mainly involves the renal tubule, accounting for clinical manifestations varying from Fanconi’s syndrome to acute tubular necrosis. Recent reports have also linked TDF-based HAART regimens to glomerular injury, characterized by mild and time-dependent elevations in serum creatinine [25]. In a study carried out by Labarga [28] entitled “Kidney tubular abnormalities in the absence of impaired glomerular function in HIV patients treated with Tenofovir”, HIV patients on TDF had higher prevalence of tubular damage compared to those on other ART as well as among the ART-naïve individuals. Tenofovir has been shown to cause renal tubular cell toxicity by impairing mitochondrial function, interfering with tubular transport, increasing oxidative stress, or forming free radicals. Our results may be contrary to Labarga’s findings because of the small number of patients on Tenofovir.

There was no statistically significant difference in mean creatinine between the DOTS and HAART/DOTS group at the intensive and the continuation phase. This result tallies with the work of Dinesh and colleagues [29], in which they were able to determine that, serum level of creatinine was normal before and after anti-tuberculosis treatment. This may be because Streptomycin was not used in the patients enrolled in our study. One of the side effects of Streptomycin is nephrotoxicity. This may explain why the level of serum creatinine in patients on combined HAART/DOTS group and DOTS group was normal. Contrary to previous studies, the incidence of rifampicin nephrotoxicity varies from 1.8–16 % of all acute renal failure. Glomerulonephritis presenting as proteinuria with acute onset deterioration of renal functions may occur [26]. The duration of therapy seems important and cases have been reported after two months of therapy; although reactions as early as 13 days have also been seen [26]. The possibility of nephrotoxic interaction between isoniazid and pyrazinamide also exists [26]. Chia-Hao and colleagues [5] also reported a 7.1 % prevalence of AKI in patients on anti-TB treatment. Within two months of intensive anti-TB treatment, 4.3 % developed AKI with 0.8 % of them reporting a prior history of rifampin exposure [5]. Our results were contrary to those of Chia-Hao and colleagues [5] because they worked on an aging population while we concentrated on an adult population with a mean age of 38.04 (±10.52) years.

The primary outcome of the present study was renal dysfunction. HIV treatment drugs are associated with decline eGFR below 60 ml/min/1.73 m^2^ and elevated serum creatinine values. Nephrotoxicity resulting from these abnormal biochemical parameters were the secondary outcomes. These drugs fight the progression of HIV and TB but pose a challenge to the kidney function of those receiving them. However, our results show very high prevalence of kidney disease in the 3 treatment groups. This is contrary to what we had estimated in our power calculation.

This study has limitations, particularly related to the cross-sectional design and the small sample size in the three treatment groups. Also, patients on treatment for HIV and tuberculosis had been on treatment for different lengths of time. In addition, measures of renal function were not repeated after three months to determine if kidney disease was acute or chronic. Also, urine microalbumin was not performed and spectrophotometric analysis could be influenced by temperature, pH, impurities and contaminants with a resultant change in the absorption properties of the sample leading to inaccurate readings.

## Conclusion

Compared to adults on DOTS, adults on HAART were more likely to have renal dysfunction (eGFR <60 ml/min per 1.73 m^2^). Adult on a combination of HAART and DOTS had a similar prevalence of renal dysfunction as those on HAART alone. This study showed that the use of the HAART regimen (Tenofovir, Lamivudine and Efavirenz combination) among the HAART treated adults was nephrotoxic. However, other combined HAART and DOTS regimens had no nephrotoxic effect. Abnormal kidney function can be associated with HAART use. The DOTS were less nephrotoxic and some of the drugs such as rifamycin reduced the nephrotoxicity of HAART. It is possible to suggest that less toxic regimens should be used in the management of HIV/AIDS and TB co-infected patients.

## Abbreviations

ATT: Anti-tuberculosis therapy
DOTS: Directly observed therapy, short-course
eGFR: Estimated glomerular filtration rate
HAART: Highly active anti-retroviral therapy
KDIGO: Kidney disease international global outcome
MDRD: Modification of diet in renal disease
NNRTs: Non nucleoside reverse transcriptase
PLWHA: People living with HIV/AIDS
WHO: World Health Organization
aOR: Adjusted odds ratio
